# Targeting SARS-CoV-2 RNA-dependent RNA polymerase: An *in silico* drug repurposing for COVID-19

**DOI:** 10.12688/f1000research.26359.1

**Published:** 2020-09-23

**Authors:** Krishnaprasad Baby, Swastika Maity, Chetan H. Mehta, Akhil Suresh, Usha Y. Nayak, Yogendra Nayak

**Affiliations:** 1 Department of Pharmacology, Manipal College of Pharmaceutical Sciences, Manipal Academy of Higher Education, Manipal, Karnataka, 576104, India; 2 Department of Pharmaceutics, Manipal College of Pharmaceutical Sciences, Manipal Academy of Higher Education, Manipal, Karnataka, 576104, India

**Keywords:** COVID-19, SARS-CoV-2, Docking, In silico, RNA-dependent RNA polymerase, Molecular dynamics simulation, Drug repurposing

## Abstract

**Background:** The coronavirus disease 2019 (COVID-19) pandemic, caused by severe acute respiratory syndrome coronavirus-2 (SARS-CoV-2), took more lives than combined epidemics of SARS, MERS, H1N1, and Ebola. Currently, the prevention and control of spread are the goals in COVID-19 management as there are no specific drugs to cure or vaccines available for prevention. Hence, the drug repurposing was explored by many research groups, and many target proteins have been examined. The major protease (M ^pro^), and RNA-dependent RNA polymerase (RdRp) are two target proteins in SARS-CoV-2 that have been validated and extensively studied for drug development in COVID-19. The RdRp shares a high degree of homology between those of two previously known coronaviruses, SARS-CoV and MERS-CoV.

**Methods:** In this study, the FDA approved library of drugs were docked against the active site of RdRp using Schrodinger's computer-aided drug discovery tools for *in silico* drug-repurposing.

**Results:** We have shortlisted 14 drugs from the Standard Precision docking and interaction-wise study of drug-binding with the active site on the enzyme. These drugs are antibiotics, NSAIDs, hypolipidemic, coagulant, thrombolytic, and anti-allergics. In molecular dynamics simulations, pitavastatin, ridogrel and rosoxacin displayed superior binding with the active site through ARG555 and divalent magnesium.

**Conclusion: **Pitavastatin, ridogrel and rosoxacin can be further optimized in preclinical and clinical studies to determine their possible role in COVID-19 treatment.

## Introduction

A novel disease was first discovered in late 2019 in a 41-year-old patient in China who was admitted to a health facility for severe respiratory syndrome and fever, dizziness, and cough. A new strain of RNA virus of the Coronaviridae family was isolated from the patient's bronchoalveolar lavage fluid and was originally unique initially referred to as 'WH-Human 1' coronavirus (and later known as '2019-nCoV') was. The novel virus was with 60–140 nm diameter with 9–12 nm long spikes, with the virion comparable to the corona of the sun. Viral genome analysis revealed that it is made of ~30k nucleotides, and has 89.1% nucleotide similarity to SARS-like CoV (genus Betacoronavirus, subgenus Sarbecovirus) ^1^. The human CoV B814-strain was isolated in 1965 from the nasal discharge of a patient with a common cold. Since then, 30 specific strains were identified. Seven hCoVs have been reported previously causes disease, including HCoV-229E, HCoV-OC43, HCoV-NL63, HCoV-HKU1, SARS-CoV, MERS-CoV, and 2019-nCoV (now established as SARS-CoV-2) ^2^. SARS-CoV emerged during the period 2000–2004, and it was discovered that the infection was descended from animals and bats as intermediate hosts ^3^. MERS-CoV was diagnosed for the first time in Saudi Arabia ^4^. The World Health Organization (WHO) named the disease coronavirus disease 2019 (COVID-19) on 12 January 2020. The same outbreak was declared a public health emergency of international concern by the WHO on 30-January-2020 due to rapid transmission mortality rate ^5^. The International Committee on the Taxonomy of Viruses Coronavirus Study Group (CSG) proposed the name of nCoV as SARS-CoV-2 ^6^.

Currently, clinical trials have approved the use of remdesivir, favipiravir, and dexamethasone, but the infection could not be cured or prevented. The remdesivir and favipiravir are comparatively expensive than dexamethasone and not affordable for people at developing countries. Vaccine development, convalescent plasma, interferon-based therapies, small molecule medications, cell-based therapy, and monoclonal antibodies (mAbs) are the various likely future medications and pathways being investigated ^7^. The development of a drug is expensive and time-consuming with a high attrition rate, which is unacceptable in the current global emergency. Hence, there is a great interest in repurposing the existing drugs and speed-up to develop antiviral therapies ^8^. The national health commission (NHC) of the People's Republic of China recommends the use of α-interferon (IFN-α), lopinavir/ritonavir, ribavirin, chloroquine phosphate, and arbidol as antiviral therapy against SARS-CoV-2. Other agents such as inhibition of fusion/entry (camostat mesylate, baricitinib, arbidol and chloroquine phosphate), the agents that disrupt SARS-CoV-2 replication (remdesivir, favipiravir, lopinavir/ritonavir), the agents that suppress excessive inflammatory response (corticosteroid), convalescent plasma, vaccines (inactivated vaccine, recombinant subunits vaccine, nucleic acid-based vaccine, adenoviral vector vaccine, recombinant influenza viral vector vaccine), a combination of traditional Chinese and Western medicine (ShuFeng JieDu capsules and Lianhua Qingwen capsules) are also gaining therapeutic interest ^9^.

The SARS-CoV-2 genetic material is translated inside the host cell to two distinct groups of proteins; structural proteins, such as Spike (S), Nucleocapsid (N), Matrix (M) and Envelope (E), and non-structural proteins (nsp) such as RNA dependent RNA polymerase (nsp12), helicase (nsp13), papain-like protease (PL ^pro ^or nsp3) and main protease M ^pro^ (also known nsp5 or 3CL ^pro^) ^10– 12^. SARS-CoV-2 replication is facilitated through a multi-subunit replication/transcription complex of non-structural viral proteins. The key aspect of the nsp complex is an RNA-dependent RNA polymerase (RdRp; nsp12). The central RdRp domain is divided into three subdomains, the thumb, palm, and right-handed cup-like fingers, which is less than 500 long amino acids length ^11^. A high level of homology has been found between SARS-CoV-2, MERS-CoV, and SARS-CoV RdRp ^13^. The RdRp requires extra factors such as nsp7 and nsp8, for its activity ^14^. Remdesivir, which was recently approved by US-FDA for COVID-19, inhibits RdRp ^15^. Furthermore, researchers have explored RdRp-target for *in silico* repurposing of drugs. Hence, this proves that Nsp12 or RdRp is an attractive target for inhibiting viral replication. In the current study, we assessed USFDA approved drugs to impede the RdRp activity of SARS-CoV-2 through an *in silico* drug-repurposing strategy.

## Methods

### Computational simulations

All computational simulation experiments were conducted on the Schrödinger Suite's (version 2020-2) Maestro graphical user interface ( www.schrodinger.com; v12.3) on an Ubuntu software desktop workstation, Intel ® Xenon ® Gold 6130 CPU @ 2.10 GHz × 64 processors, Quadro P620 / PCle / SSE2 graphics card and 134.8 GB RAM. Alternatively, free software including Autodock 4.2.6, Gromacs 5.1, ArgusLab 4.0 and PDB2PQR 3.0 could be used for such study.

### Ligand preparation

Molecules licensed by US-FDA were downloaded from https://www.drugbank.ca (date of access: 15 Feb 2020). The molecules were configured using LigPrep, wherein the molecules generated the 3D coordinates ^16^. Using the Epik module, the suitable ionization state was predicted at pH 8.0, types of tautomers were produced, and proper chirality was estimated. Finally, the molecules' structure was minimized with energy using the OPLS3e force field ^17^. Alternatively, the Zinc database can be used for FDA approved drug and ArgusLab 4.0, PDB2PQR can be used for ligand configuration and stabilising the ligand state at certain pH respectively.

### Protein preparation

The electron microscopical RdRp coordinate structure of SARS-CoV-2 linked to template-primer RNA which was 50- base pair long and remdesivir triphosphate was downloaded from Protein Data Bank (PDB), accession code 7BV2, at a resolution of 2.5 Å. The RdRp-NS7-NS8 complex was linked to three Mg ^2+^ ions (coordinated by Asp760, Asp761, Asp618, and pyrophosphate), double-stranded RNA (14-base template strand and 11-base primary strand) and monophosphate remdesivir (RMP). The Protein Prep Wizard was used for the optimization of protein structure ^18^. ArgusLAb 4.0 software, which is free tool for protein preparation for docking studies can be used for the same. In this process, the missing hydrogens, side chains, and other organic solvent molecules and water residues were eliminated. The proper ionization state was then created at pH 8.0 using PROPKA3, regenerating the hydrogen bond network, and finally minimizing the protein structure using a restrained minimization technique ^19, 20^.

### Ligand docking

Ligand docking of molecules licensed by US-FDA (~2800 Nos) was performed using the Schrodinger Glide v8.7 module ^21^. Alternatively, AutoDock 4.2.6, an open access package for ligand docking, can be used for the same function. The binding site of the ligands on the protein was assessed using the Receptor Grid Generation tool using the centroid formed around the bound ligand. The docking was initially performed using high-throughput virtual screening (HTVS). Later, top molecules with strong binding to the active site of RdRp and docking scores were moved ahead for standard precision (SP)-docking. The single best pose of each molecule was saved during ligand docking ^22^.

### Molecular dynamics (MD) simulation

The MD-simulation was run on Schrodinger's Desmond module ^23^. Alternatively, the free software NAMD with VMD molecular simulation could be used. The solvated water-soaked system was generated using the Desmond System Builder tool. The solvating system used in the experiment was the TIP3P model. An orthorhombic simulation was a box that generated at least 10 Å from the outer surface of the protein with periodic boundary conditions. The system was neutralized by adding a reasonable amount of counterions. By adding 0.15 M NaCl into the simulation panel, the isosmotic condition was conserved. A pre-defined equilibration procedure was performed before the simulation. The MD simulation was performed at an ambient pressure of 1.013 bar, and a temperature of 300°K, with 1000 frames saved to the trajectory, for 50 nsec period. Later the simulation was analyzed using a simulation interaction diagram, wherein protein-ligand root mean square deviation (RMSD), protein root mean square fluctuations (RMSF), ligand RMSF, protein-ligand contacts, ligand-protein contacts, ligand torsion profile. were analyzed.

## Results and discussion

### Structure of RdRp

SARS-CoV-2 RdRp is a central polymerase involved in the replication of RNA, is a big enzyme consisting of 932 amino acids. The amino acid framework of SARS-CoV RdRp linked to the Nsp7 and Nsp8 cofactors ^24^. Structurally, the RdRp protein is divided into the N-terminal and polymerase domains. The N-terminal domain extends from amino acid residues 1 to 397. The polymerase domain extends from amino acids 398 to 919 and is similar to the previously reported SARS-CoV nsp12 (PDB ID: 6NUR). The RdRp polymerase domain is subdivided into three structurally different subunits, namely the finger subunit (398–581 and 628–687 amino acids), palm subunit (582–627 and 688–815 amino acids), and thumb subunit (816–919 amino acids), which embraces a "right-handed" shaped conformation ^15^. The active site is located at the hinge interface between the finger and thumb subunits of the SARS-CoV-2 RdRp, which is an evolutionary conserved in both the coronaviruses reported earlier, ie. MERS and SARS-nsp12. The active site of nsp12 is situated in the middle of the substrate domain where the synthesis of RNA takes place as an RNA template is accessed from the template input channel and nucleoside triphosphate (NTP) from NTP input channel ^25^. Structural assessment of SARS-CoV-2 defined in RdRp displayed the presence of seven retained motifs (A-G motifs), which play a key role in the association of substrate and nucleotides. They also play a key role in catalysis of the attachment of the NTP. Antiviral drugs function by binding to first replicated base pairs and thereby stopping the chain elongation cycle. The sensitivity of the RdRp binding to the RNA prototype has been enhanced by the interaction of the nsp7 and nsp8 co-factors with RdRp. The RNA polymerization activity appeared to be impaired in the presence of remdesivir active triphosphate (RTP), which is being investigated in clinical trials. Remdesivir blocked the polymerization process of RdRp at 1 mM and 10 mM ATP completely. However, remdesivir alone on its own or in its monophosphate form did not demonstrate activity at 5 mM concentration. Remdesivir monophosphate was found to be covalently linked to the primary strand in protein structure to the pyrophosphate moiety and three magnesium as catalytic ions. The ligand-protein interaction of remdesivir also has two hydrogen bonds, with the uridine base, which leads to a stable complex formation. Predominant interactions include those with K545 and R55 side chains, the interaction of magnesium ions with the backbone of the phosphate diester, as well as with the primary strand RNA. Seven conserved motifs from A to G represent the nsp12 RdRp catalytic active site, among which the palm subdomain with an SDD sequence (residues 759-761) in motif C, including amino acid residues D760 and D761 in the coordination of the two magnesium ions. The template-primer RNA, which oriented in the same fashion as template-primer RNA in the poliovirus RdRp elongation complex and residues involved in binding as well as interactions to 2′-OH groups, was found to be highly conserved ^15^.

### HTVS and SP Docking results

**Figure 1.**
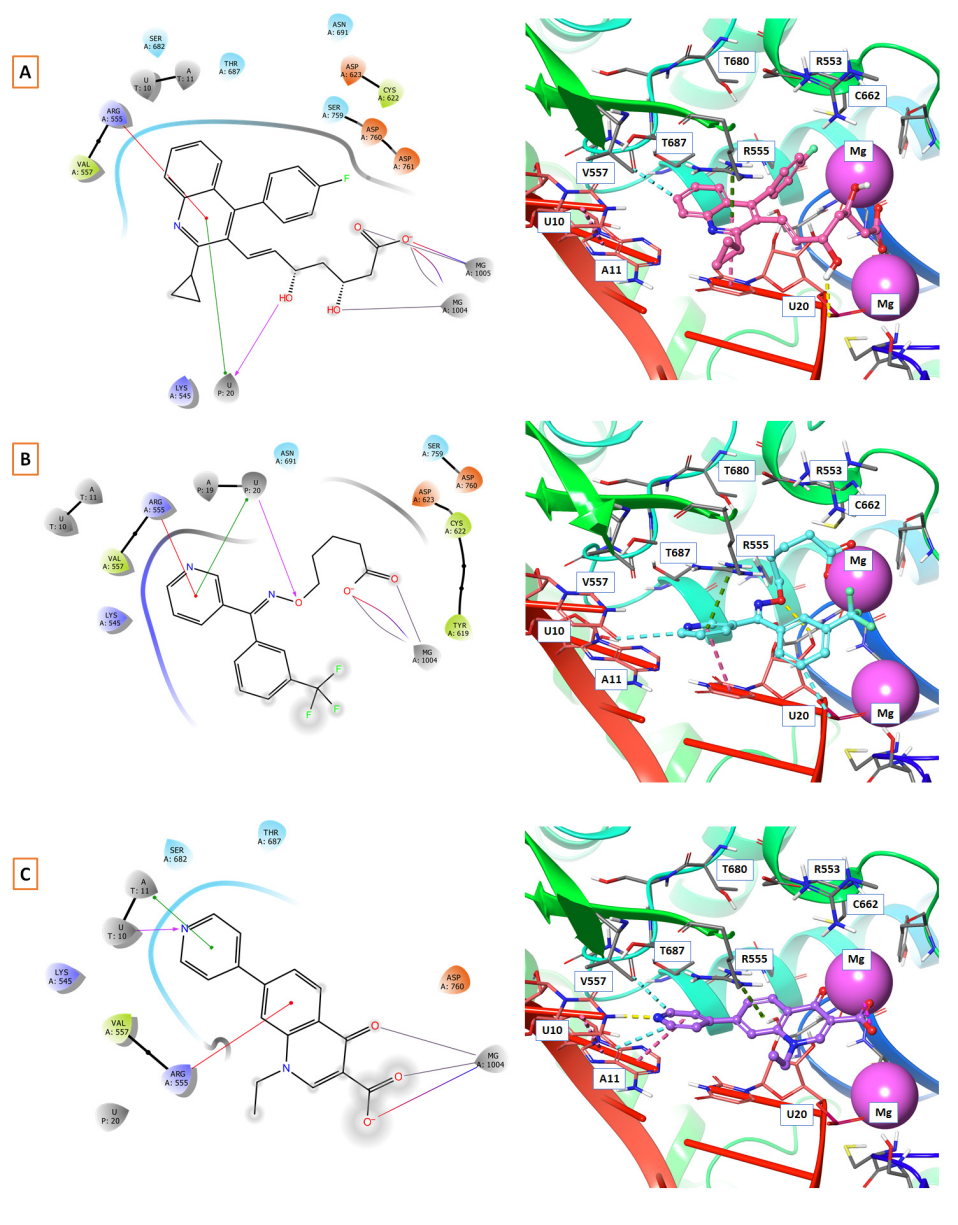
2D and 3D Binding mode. Shown are ( **A**) pitavastatin, ( **B**) ridogrel, and ( **C**) rosoxacin with SARS-CoV-2 RdRp.

The docking provides details about binding affinity and orientation of the interactions between ligand and protein. HTVS was initially performed using ~2800 drug molecules. The HTVS docking is used to screen large numbers of ligands rapidly. However, HTVS seems to have more limited conformational sampling than docking with SP, and hence HTVS can not be used with ranking in place. Hence, molecules were advanced to the higher precision of docking known as SP. The SP docking was performed on 500 molecules, which exhibited good interactions with the RdRp and had topped in the docking score. Among the 500 molecules, 14 displaying top SP-docking scores and the conserved interactions with the active site as that of remdesivir were shortlisted based on visual inspection ( Table 1). These molecules were able to interact with RdRp amino acid residues via a predominant metal coordination bond and hydrogen bonding with the active site. The interactions exhibited by rosoxacin, kappadione, tedizolid phosphate, levomefolic acid, etodolac, pitavastatin, montelukast, norfloxacin, cinoxacin, mefenamic acid, diflunisal, fospropofol, fluvastatin, and ridogrel was found to be prominent. Figure 1 depicts the binding mode of Pitavastatin, Ridogrel and Rosoxacin, which emerged as the best molecule by MD simulations. 

**Table 1.** Intermolecular interactions of shortlisted drugs and the SARS-CoV-2 RdRp.

Drug/Drug-Bank ID/ Structure	SP- Docking Scores	π- π stacking	π- cation interactions	H- bonding	Metal Coordination Bond	Salt bridges	Use
1. Rosoxacin DB00817 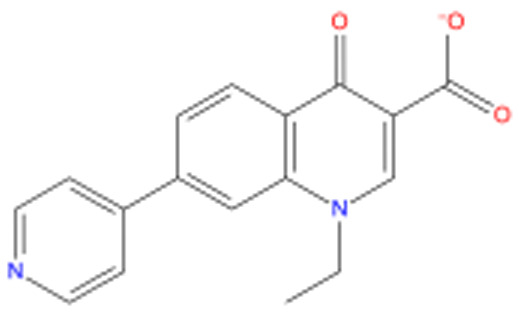	-10.263	A T:11	ARG 555	U T:10	MG A: 1004	MG A: 1004	Quinolone antibiotic for Respiratory, urinary, GI tract, infection
2. Levomefolic acid DB11256 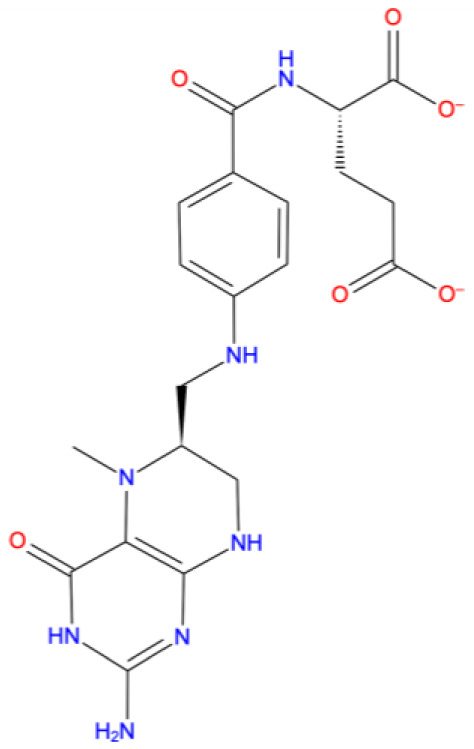	-10.099	U P: 20	ARG555	-	MG:A 1004	ARG553 LYS621	Metabolite of folic acid (Vitamin B9), used as folate supplement
3. Etodolac DB00749 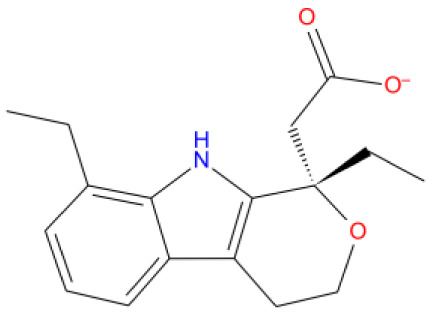	-9.473	U P: 20	ARG555	-	MG:A 1004	MG:A 1004	NSAIDs, in osteoarthritis and rheumatoid arthritis.
4. Kappadione DB09332 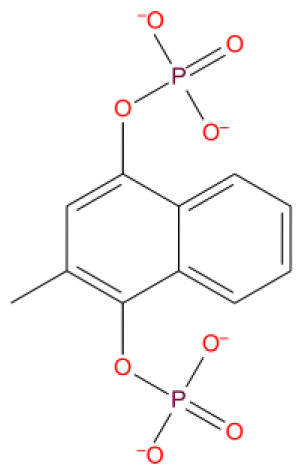	-9.380	-	ARG555 U P:20	ARG555 U T:10	LYS545	LYS545 ARG555 MG:A 1004	Vitamin K derivative, a coagulant used to prevent hemorrhagic disease in newborns
5. Pitavastatin DB08860 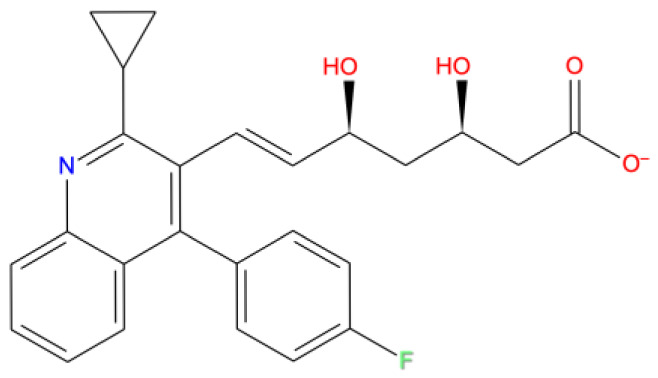	-9.038	U P: 20	ARG555	U P: 20	MG:A 1004 MG A:1004	MG A: 1005	Statin, lipid lowering drug for Primary hyperlipidemia or mixed dyslipidemia
6. Montelukast DB00471 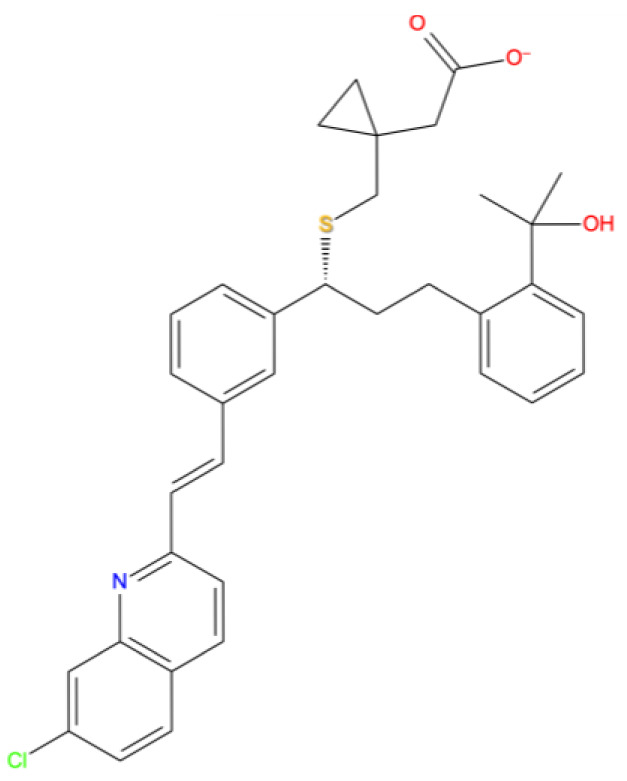	-8.909	U P: 20 MG: 1005	ARG555 (2)	-	MG A: 1004	MG A: 1004	Leukotriene receptor antagonist, chronic asthma, exercise induced asthma, seasonal allergic rhinitis
7. Fluvastatin DB01095 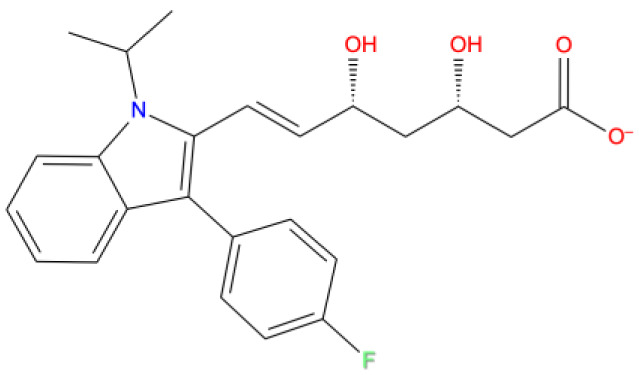	-8.863	U P: 20	-	-	MG:A 1004	MG:A 1005	Statin, hypolipidemic in cardiovascular patient
8. Norfloxacin DB01059 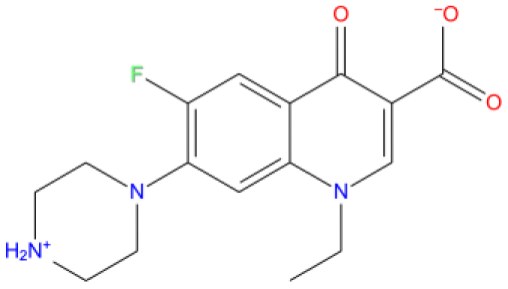	-8.735	-	U T: 10 ARG555	SER682 U T: 10	MG A: 1004	MG A: 1004 (2)	A synthetic fluoroquinolone, used in UTI
9. Cinoxacin DB00827 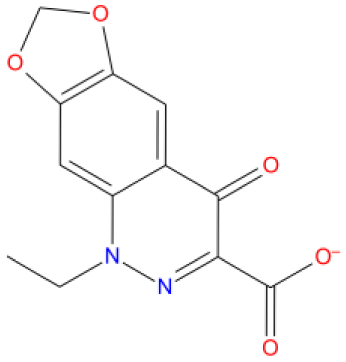	-8.717	-	ARG555	-	MG:A 1004	MG:A 1004	Synthetic antimicrobial related to oxolinic acid, used in UTI
10. Tedizolid Phosphate DB14569 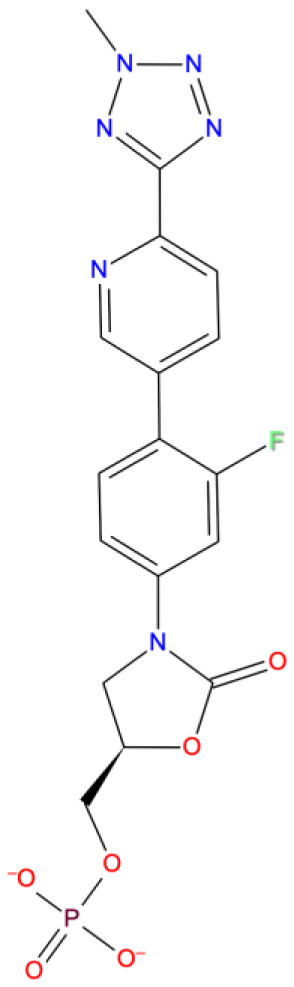	-8.578	A T:11 U P:20	-	U T:10	MG A:1004 MG A: 1005	-	Oxazolidinone class of antibiotics; indicated for the treatment of skin infections
11. Mefenamic acid DB00784 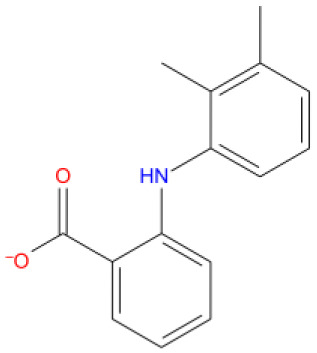	-8.358	U P: 20	-	-	MG A:1004	MG A: 1004	NSAIDs, in rheumatoid arthritis, osteoarthritis, dysmenorrhea, and mild to moderate pain, inflammation, and fever.
12. Diflunisal DB00861 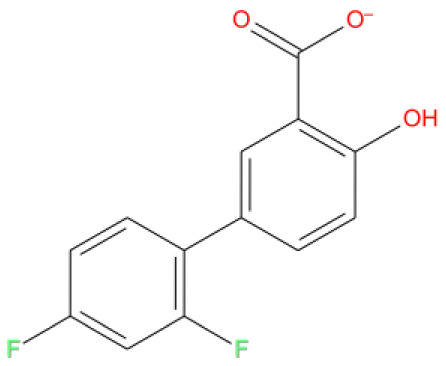	-8.328	U P: 20	ARG555	-	MG:A 1004	MG:A 1004	NSAIDs, a salicylate derivative, in mild to moderate pain
13. Fospropofol DB06716 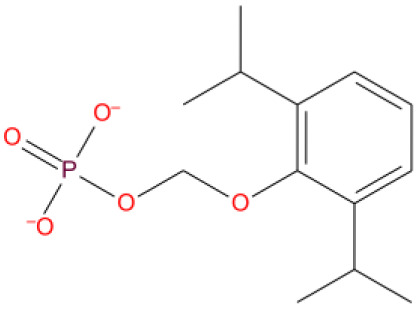	-8.325	U P: 20	ARG555	-	MG:A 1004	MG:A 1004	Prodrug, converted to propofol in liver, short acting hypnotic/sedative/ anesthetic agent
14. Ridogrel DB01207 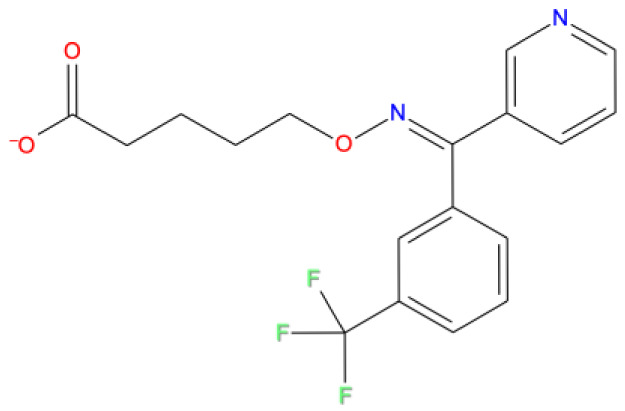	-8.321	U P:20	ARG555	U:P:20	MG:A 1004	MG:A 1004	Thromboxane synthase inhibitor in acute myocardial infarction. Aspirin overtook the use

The mode of interaction of selected molecules with the RdRp active site in the SP-docking is depicted in Table 1. The shortlisted molecules interact with conserved residue ARG555 and divalent magnesium in the manner Remdesivir interacted with RdRp. Drugs including rosoxacin, levomefolic acid, etodolac, kappadione, pitavastatin, montelukast, norfloxacin, cinoxacin, diflunisal, fospropofol and ridogrel have demonstrated π-cation interactions with ARG555. Norfloxacin and cinoxacin have demonstrated π-π stacking interactions with 20 unspecified residues: (U P: 20). In SP-docking, the drugs shortlisted made metal coordination and salt bridges with MG A:1004 and MG A:1005 (divalent magnesium ions) in the same way remdesivir was bound ( Table 1). Rosoxacin, pitavastatin and ridogrel had strong interactions across metal coordination bonds among the molecules shortlisted by SP-docking, and π-cation with RdRp can be further tested for their *in vitro* function.

### MD simulations

**Figure 2.**
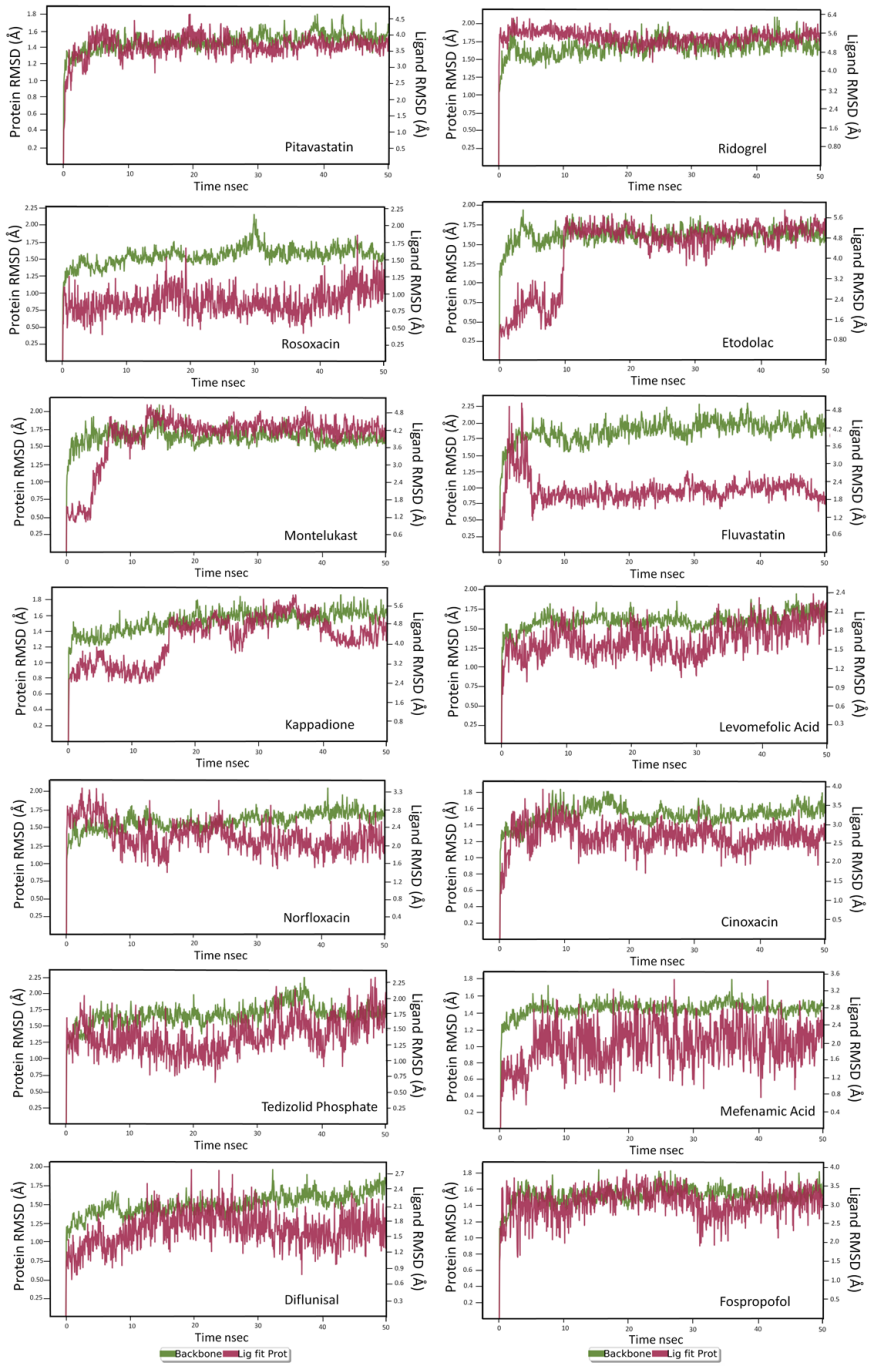
Route mean square deviations (RMSD) plot of RdRp protein-ligand interactions of shortlisted 14 molecules.

The RdRp-protein and ligand binding stability were checked based on the RMSD fluctuations during MD-simulations. In the MD simulation, the RMSD fluctuation was measured individually for the protein and ligand structures, and it falls within 3 Å. Hence the complex was considered to be stable. Besides, the persistence of the intermolecular interactions between ligand and RdRp was also monitored during the simulation. The analysis RMSD plot for the structures saved in the trajectory generated during the MD-simulation exhibited stable bindings for 14 drug molecules. For rosaxocin, pitavastatin and ridogrel in complex with the RdRp-protein, the RMSD fluctuations for the ligand and protein remained within 2.0 Å. Figure 2 depicts the ligand and protein RMSD plot for the shortlisted 14 drugs.

### Pitavastatin

**Figure 3.**
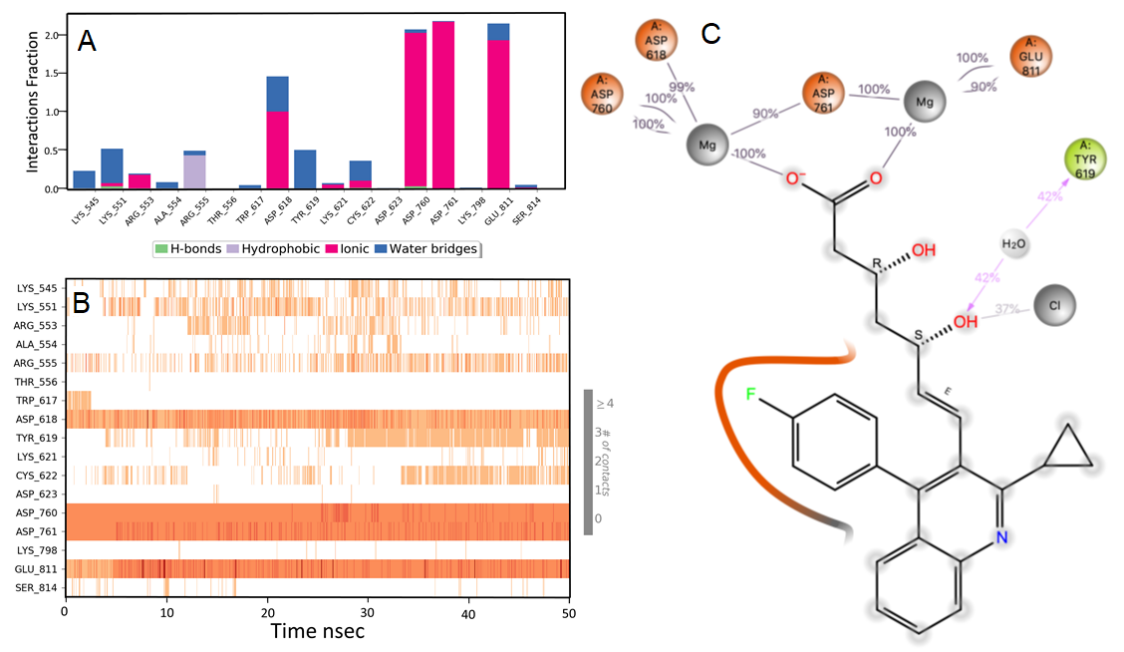
Interaction diagram of pitavastatin with RdRp protein observed during the molecular dynamics simulation. ( **A**) The protein-ligand interaction diagram. ( **B**) The residues that interact with the ligand in each trajectory frame. The residues making more than one contact are shown in darker color shade. ( **C**) Schematic diagram of ligand interaction with the amino acid residues of protein during MD simulation. Interactions that occur more than 30% of the simulation time are shown.

During the initial phase of MD simulations, the RdRp-bound pitavastatin complex exhibited higher fluctuations due to the equilibration. For the initial 10 nsec of the simulation, the protein backbone exhibited higher RMSD fluctuation. The latter 40 nsec the protein backbone fluctuations remained within the range of 0.6 Å, indicating stabilization of the protein structure. Similarly, the ligand showed consistently stable interactions throughout the simulation. The ligand RMSD fluctuations remained within the range of 0.5 Å for the remaining 40 nsec of the simulation indicated a stable ligand in the complex ( Figure 2). The interaction with residues ASP618, ASP760, ASP761, and GLU811 exhibited maximum contacts with the ligand during MD simulation. The bridged hydrogen bonding was exhibited with the ligand was amino acid residue TYR619 over more than 30.0% of the simulation time in the 50 nsec trajectory. A significant metal coordination bond was formed with the divalent magnesium and residues ASP618, ASP760, ASP761, and GLU811. The majority of the interactions observed between the protein and ligand during the docking were consistently retained during the MD simulation ( Figure 3). Pitavastatin is used as a primary hyperlipidemic agent and in mixed dyslipidemia. It lowers the elevated total cholesterol, low-density lipoprotein, apolipoprotein-B, triglycerides, and increases high-density lipoprotein. Statins are commonly prescribed in cardiovascular patients. Statins offer protection to vasculatures and are known to have pleiotropic effects in the body, especially in modulating the inflammatory process at the cellular level ^26^. One of the reports on *in silico* studies analysed the statins interaction with M ^pro^, and they have also reported the pitavastatin as a hit-drug ^27^. These combined M ^pro^ and RdRp inhibitory properties of statins might help to tackle COVID-19.

### Ridogrel

**Figure 4.**
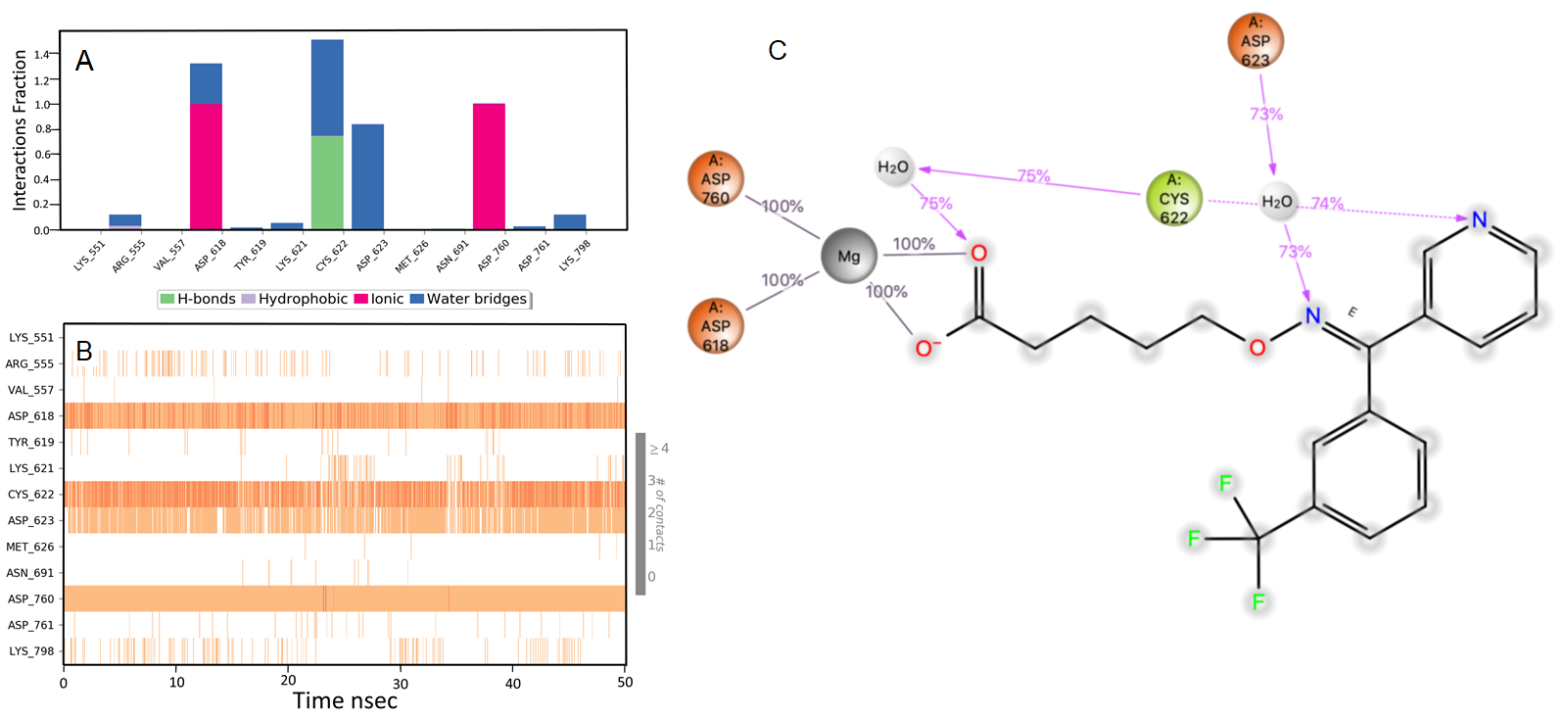
Interaction diagram of ridogrel with RdRp protein observed during the molecular dynamics simulation. ( **A**) The protein-ligand interaction diagram. ( **B**) The residues that interact with the ligand in each trajectory frame. The residues making more than one contact are shown in darker color shade. ( **C**) Schematic diagram of ligand interaction with the amino acid residues of protein during MD simulation. Interactions that occur more than 30% of the simulation time are shown.

The RdRp bound ridogrel complex exhibited a mixture of hydrophilic and ionic interactions during the MD simulation. For the initial ten nsec of the simulation, the protein backbone exhibited higher RMSD fluctuation. The latter 40 nsec the protein backbone fluctuations remained within the range of 0.6 Å, indicating stabilization of the protein structure ( Figure 2). Similarly, the ligand showed a higher fluctuation for the initial 15 nsec of the simulation. The ligand RMSD fluctuations remained within the range of 1.5 Å for the remaining 35 nsec of the simulation, indicating a relatively stable ligand-protein complex. After the initial fluctuation for a period of 10 nsec due to the equilibration, the RMSD for the protein structures remained between 2.6-4.4 Å until the end of the simulation. Amino acid residues ASP760 followed by CYS618, ASP622 and ASP623 interacted to the greatest extent with the ligand. The residues CYS622 and ASP623 made bridged hydrogen bonding interaction with the ligand which accounted to have 75 and 74% interaction with the protein residues, respectively. The amino acid residues ASP618 and ASP760 made metal coordination bonds with divalent magnesium. The majority of the interactions observed between the protein and ligand during the docking were consistently retained during the MD simulation ( Figure 4). Based on the RdRp interaction, we propose that ridogrel can be repurposed for COVID-19 as an antiviral agent. Ridogrel is a thromboxane synthase inhibitor and thromboxane or prostaglandin/endoperoxide receptor blocker. It is used for the prevention of systemic thromboembolism in acute myocardial infarction ^28^. Redogrel is been replaced with aspirin in the therapy as aspirin was found to be clinical superior. The RdRp inhibitory properties of ridogrel might make it a useful drug in COVID-19. Hence, it can be recommended for further screening and optimize it by *in vitro* assays.

### Rosoxacin

**Figure 5.**
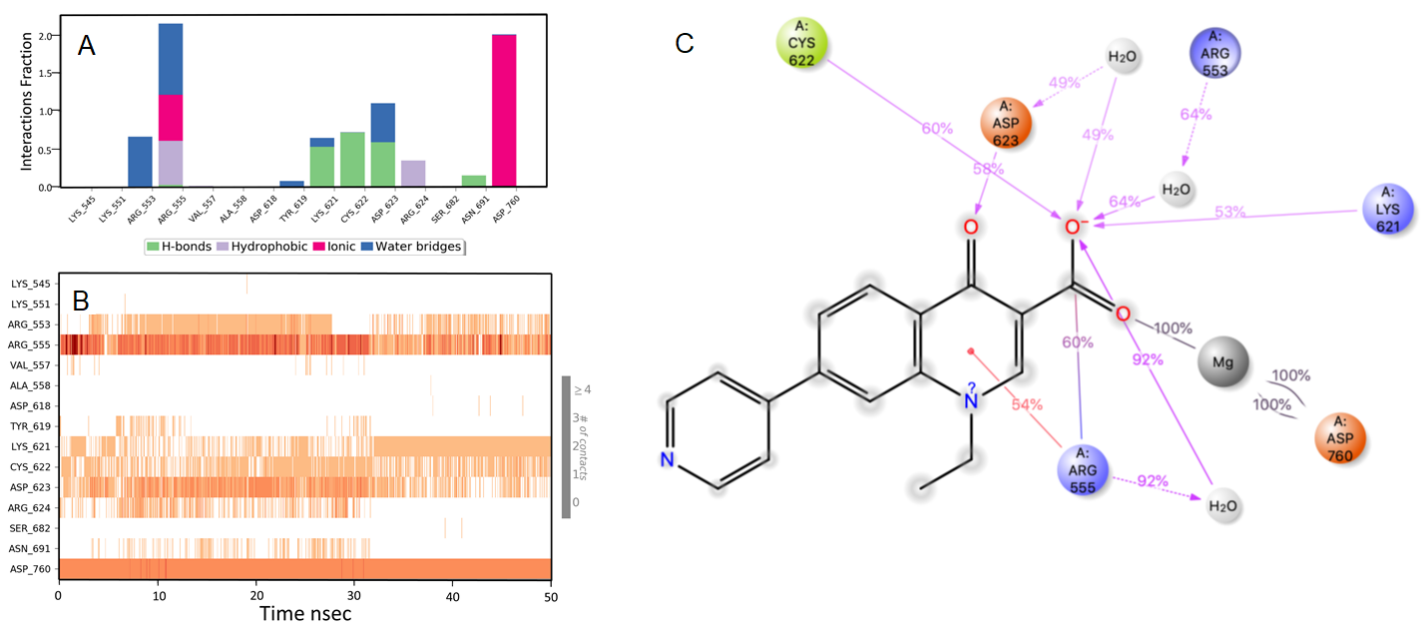
Interaction diagram of rosaxocin with RdRp protein observed during the molecular dynamics simulation. ( **A**) The protein-ligand interaction diagram. ( **B**) The residues that interact with the ligand in each trajectory frame. The residues making more than one contact are shown in darker colour shade. ( **C**) Schematic diagram of ligand interaction with the amino acid residues of protein during MD simulation. Interactions that occur more than 30% of the simulation time are shown.

RdRp bound with rosaxocin exhibited a mixture of hydrophobic interactions and hydrophilic interactions during the MD-simulation. For the initial 20 nsec of the simulation, the protein backbone exhibited higher RMSD fluctuation. The latter 30 nsec the protein backbone fluctuations remained within the range of 0.8 Å, indicating stabilization of the protein structure ( Figure 2). Similarly, the ligand showed a higher fluctuation for the initial 40 nsec of the simulation. The ligand RMSD fluctuations remained within the range of 1.4 Å for the remaining 30 nsec of the simulation indicated a stable ligand-protein complex. The residues ASP760, ARG555 and ASP623 made maximum contact with the ligand. A predominant π-cation interaction was exhibited with ARG555 residue (54%). A direct salt bridge interaction was observed with ARG555 with the carbonyl carbon, and a bridged hydrogen bonding interactions, which accounted for 92% was observed. Similarly, bridged hydrogen bonding interactions were observed with ARG553 and carboxyl electronegative oxygen (accounted for 64%). The amino acid residues LYS621, CYS622 made direct hydrogen-bonding interactions with carboxyl electronegative oxygen (accounted for 53%, 60%) and ASP623 made direct hydrogen bonding with unsaturated oxygen located in the ligand (58%)respectively over more than 30.0% of the simulation time in the 50 nsec simulation trajectory. Additionally, metal coordination interaction was observed with ASP760 with the divalent magnesium. The majority of the interactions observed between the protein and ligand during the docking were consistently retained during the MD simulation ( Figure 5). Rosoxacin is a broad-spectrum quinolone antibiotic for the treatment of urinary tract infections (UTIs) and sexually transmitted diseases ^29^. The popular anti-COVID-19 drugs chloroquine and hydroxychloroquine have the quinoline moiety ^30^. Considering the PK/PD and the safety profiles of rosoxacin, it can be taken forward in clinical trials as a repurposed candidate for COVID-19. This study is the first report of a broad-spectrum antibiotic with a high affinity for RdRp identified by *in silico* tools.

**Figure 6.**
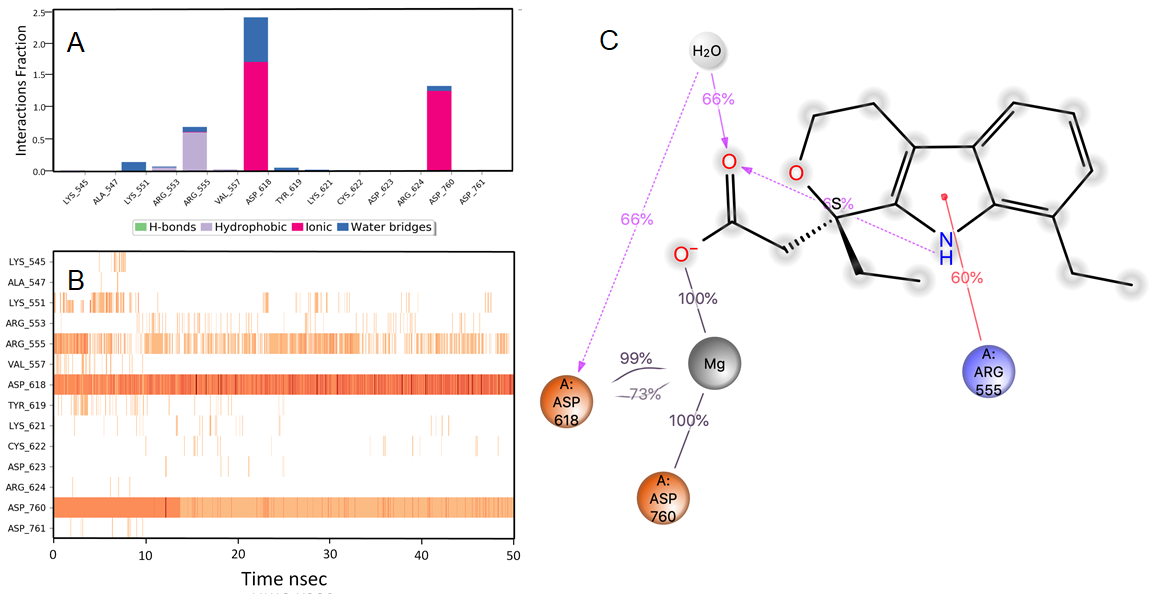
Interaction diagram of etodolac with RdRp protein observed during the molecular dynamics simulation. ( **A**) The protein-ligand interaction diagram. ( **B**) The residues that interact with the ligand in each trajectory frame. The residues making more than one contact are shown in darker color shade. ( **C**) Schematic diagram of ligand interaction with the amino acid residues of protein during MD simulation. Interactions that occur more than 30% of the simulation time are shown.

The other molecules short listed are from the diverse pharmacological group can be repurposed by studying *in vitro*. These molecules include etodolac, montelukast, fluvastatin, kappadione, levomefolic acid, norfloxacin, cinoxacin, tedizolid phosphate, mefenamic acid, diflunisal and fospropofol. The details of these MD-simulation data and molecular interaction are represented in Figure 6– Fiugre 16.

**Figure 7.**
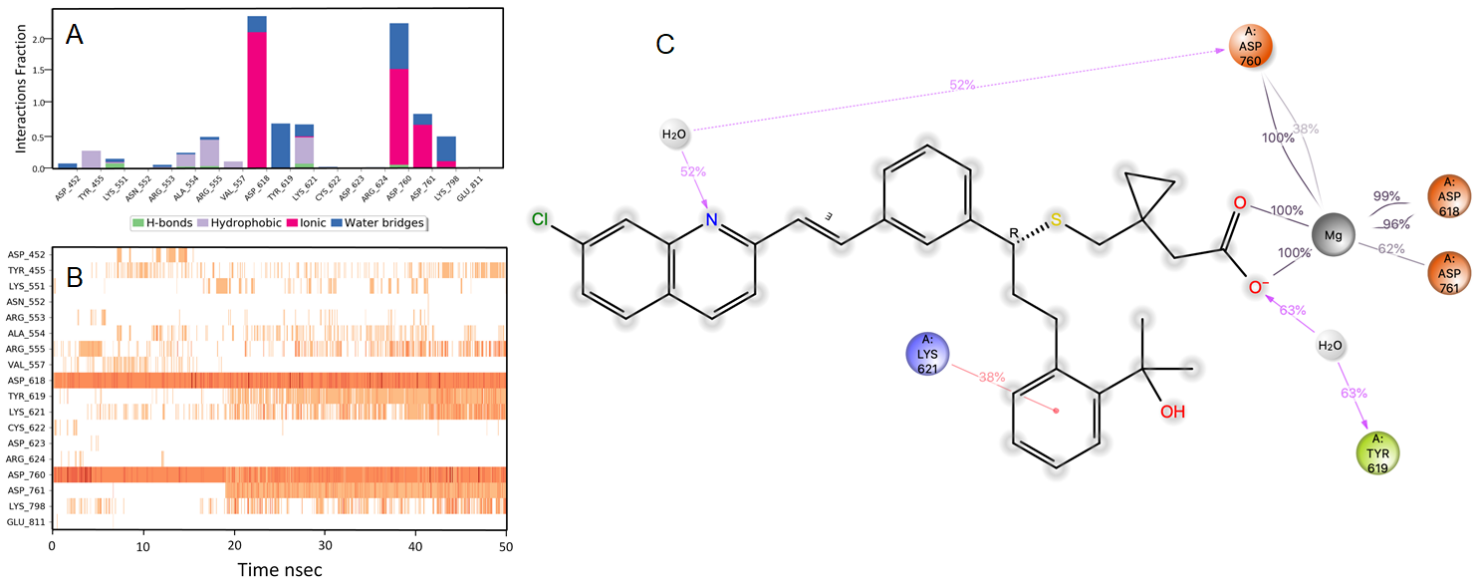
Interaction diagram of montelukast with RdRp protein observed during the molecular dynamics simulation. ( **A**) The protein-ligand interaction diagram. ( **B**) The residues that interact with the ligand in each trajectory frame. The residues making more than one contact are shown in darker color shade. ( **C**) Schematic diagram of ligand interaction with the amino acid residues of protein during MD simulation. Interactions that occur more than 30% of the simulation time are shown.

**Figure 8.**
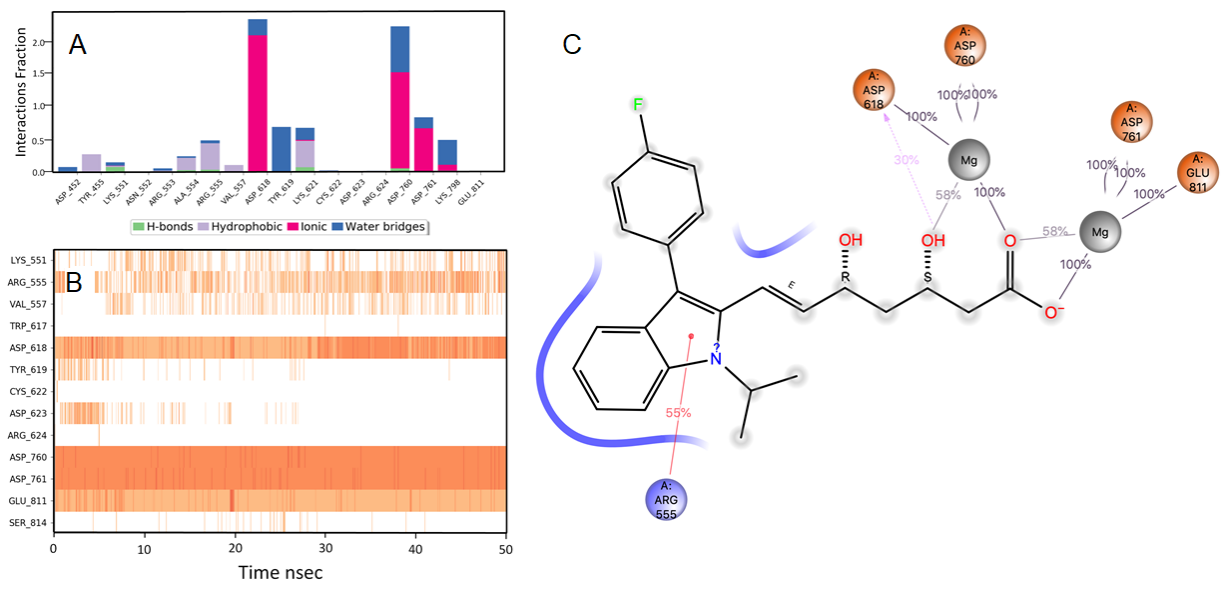
Interaction diagram of fluvastatin with RdRp protein observed during the molecular dynamics simulation. ( **A**) The protein-ligand interaction diagram. ( **B**) The residues that interact with the ligand in each trajectory frame. The residues making more than one contact are shown in darker color shade. ( **C**) Schematic diagram of ligand interaction with the amino acid residues of protein during MD simulation. Interactions that occur more than 30% of the simulation time are shown.

**Figure 9.**
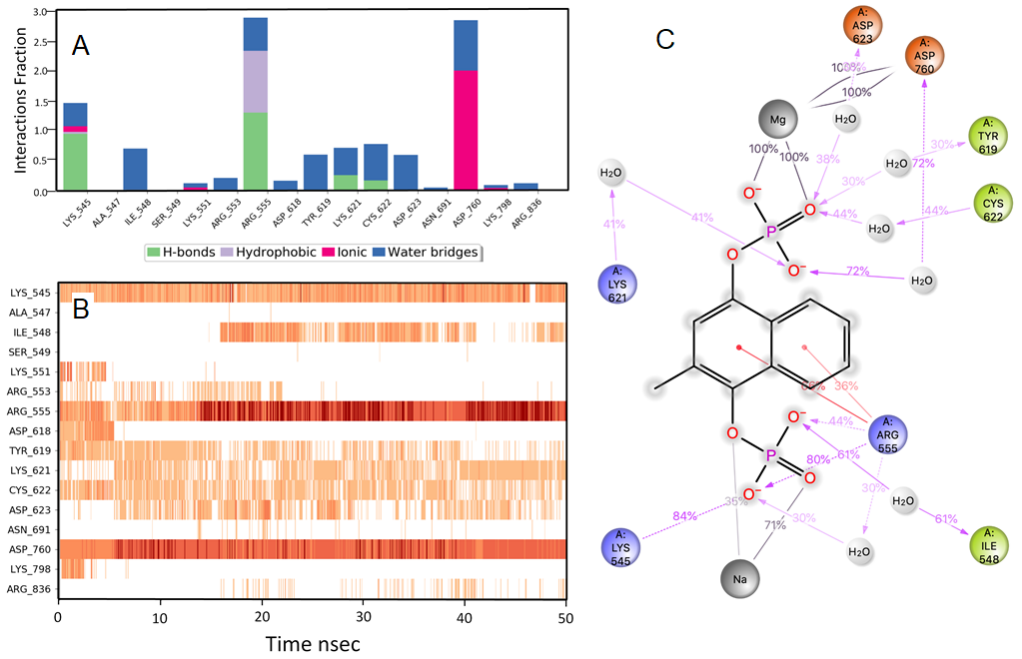
Interaction diagram of kappadione with RdRp protein observed during the molecular dynamics simulation. ( **A**) The protein-ligand interaction diagram. ( **B**) The residues that interact with the ligand in each trajectory frame. The residues making more than one contact are shown in darker color shade. ( **C**) Schematic diagram of ligand interaction with the amino acid residues of protein during MD simulation. Interactions that occur more than 30% of the simulation time are shown.

**Figure 10.**
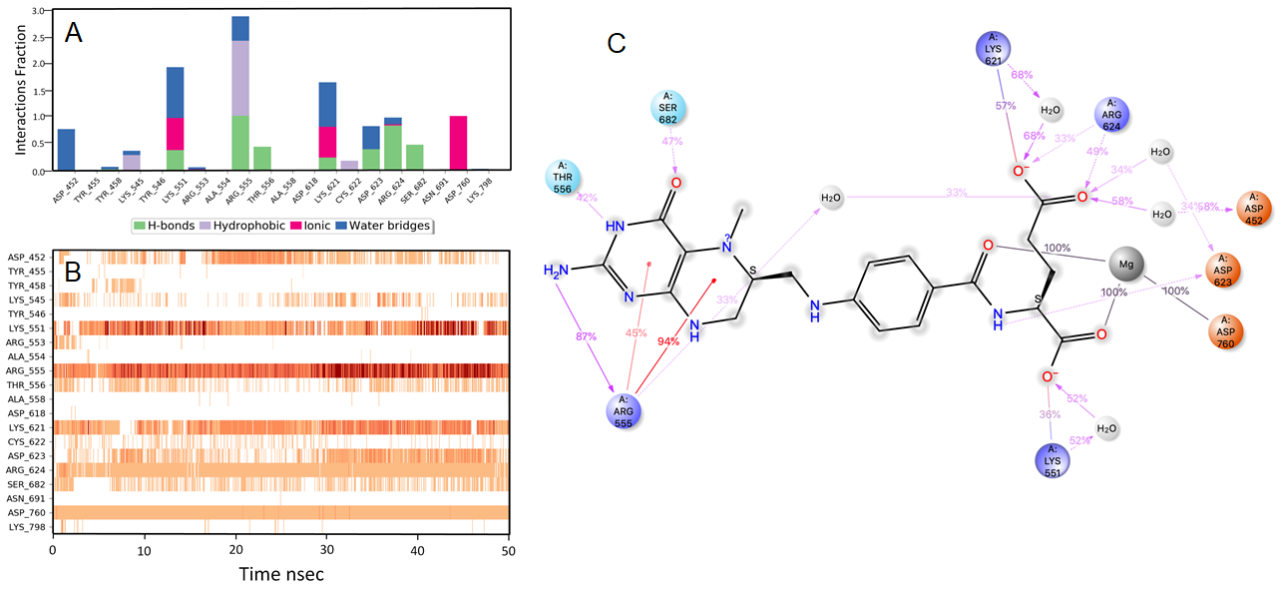
Interaction diagram of levomefolic acid with RdRp protein observed during the molecular dynamics simulation. ( **A**) The protein-ligand interaction diagram. ( **B**) The residues that interact with the ligand in each trajectory frame. The residues making more than one contact are shown in darker color shade. ( **C**) Schematic diagram of ligand interaction with the amino acid residues of protein during MD simulation. Interactions that occur more than 30% of the simulation time are show.

**Figure 11.**
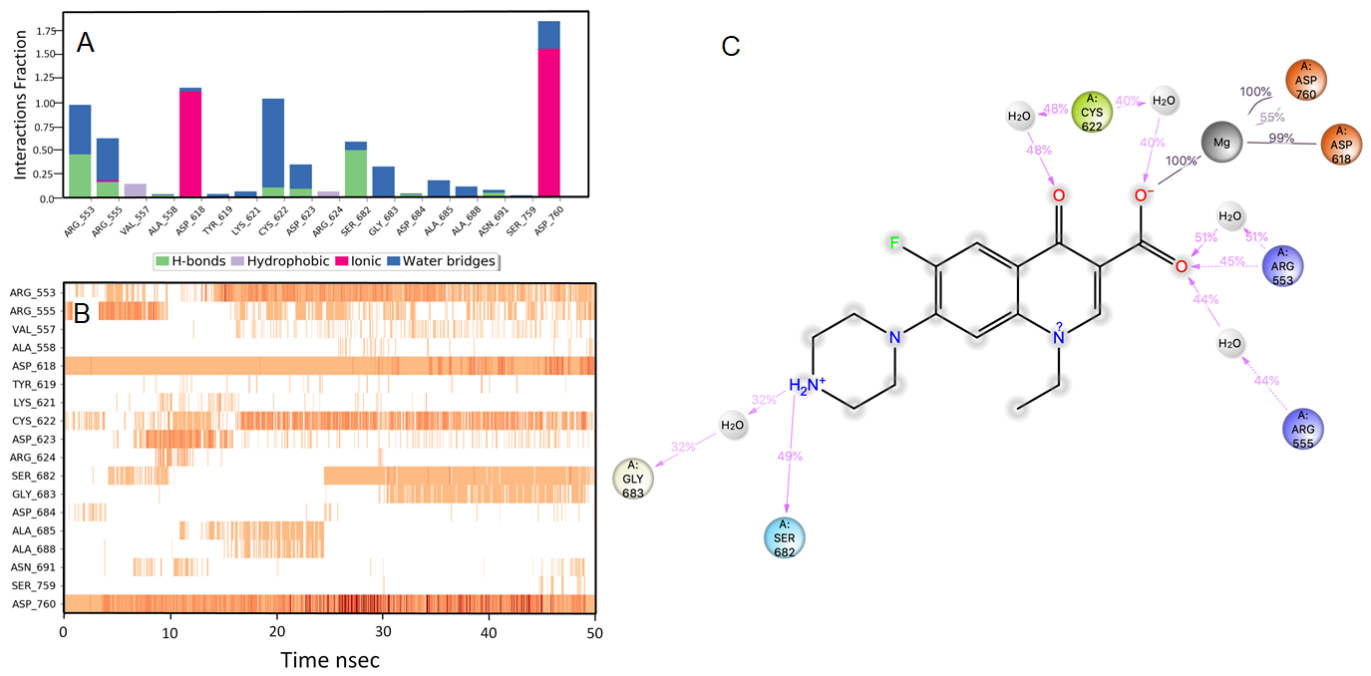
Interaction diagram of norfloxacin with RdRp protein observed during the molecular dynamics simulation. ( **A**) The protein-ligand interaction diagram. ( **B**) The residues that interact with the ligand in each trajectory frame. The residues making more than one contact are shown in darker color shade. ( **C**) Schematic diagram of ligand interaction with the amino acid residues of protein during MD simulation. Interactions that occur more than 30% of the simulation time are shown.

**Figure 12.**
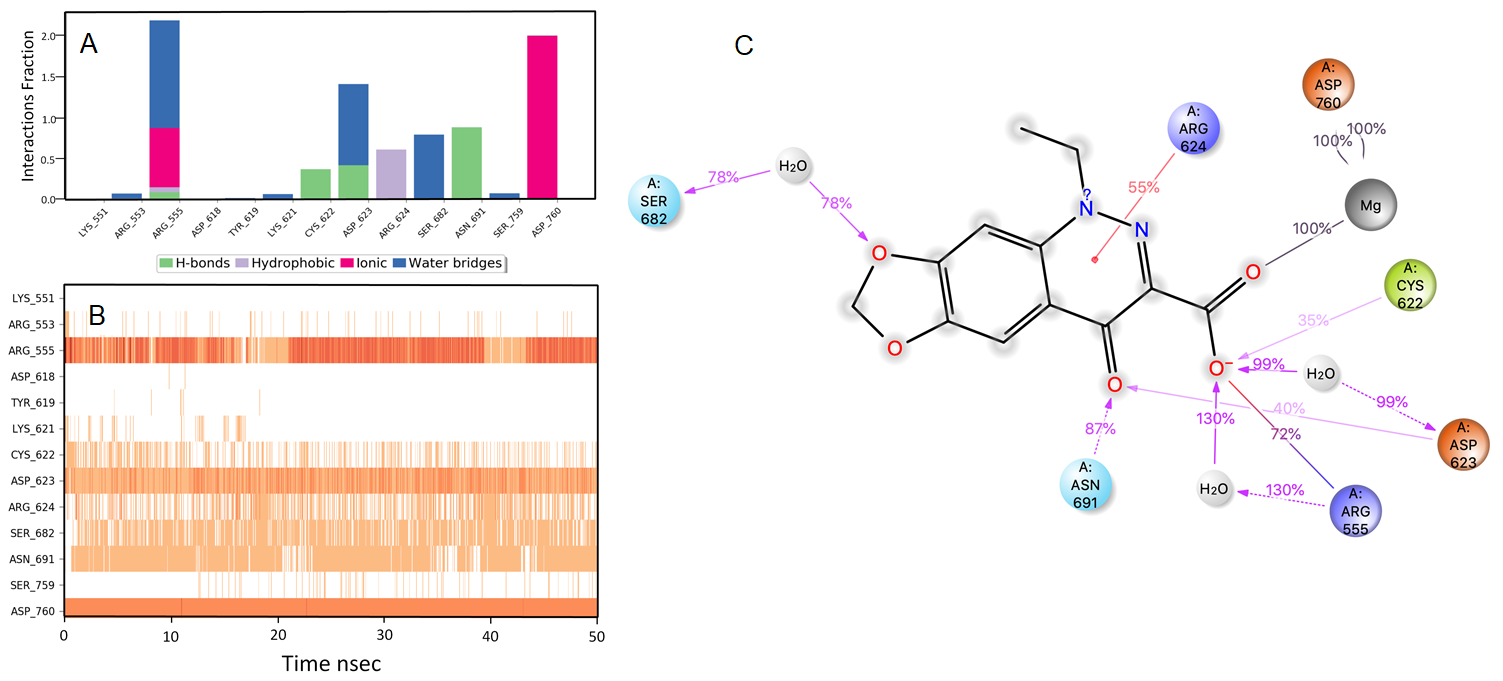
Interaction diagram of cinoxacin with RdRp protein observed during the molecular dynamics simulation. ( **A**) The protein-ligand interaction diagram. ( **B**) The residues that interact with the ligand in each trajectory frame. The residues making more than one contact are shown in darker color shade. ( **C**) Schematic diagram of ligand interaction with the amino acid residues of protein during MD simulation. Interactions that occur more than 30% of the simulation time are shown.

**Figure 13.**
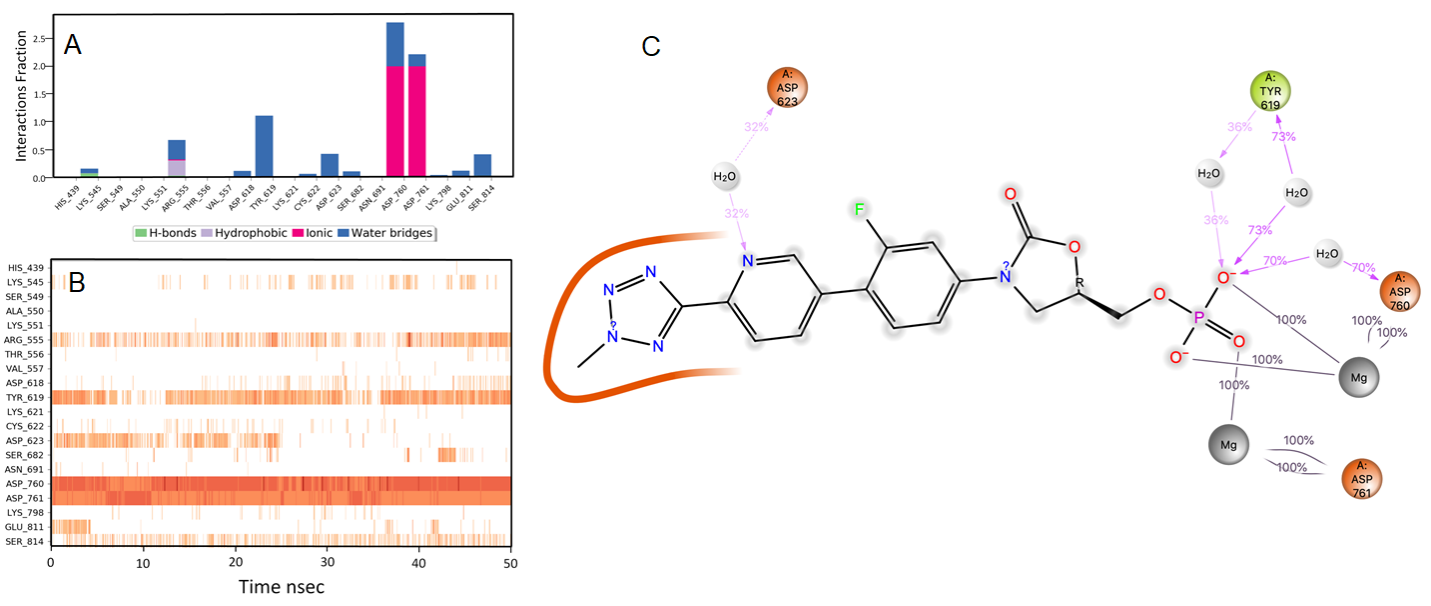
Interaction diagram of tedizolid phosphate with RdRp protein observed during the molecular dynamics simulation. ( **A**) The protein-ligand interaction diagram. ( **B**) The residues that interact with the ligand in each trajectory frame. The residues making more than one contact are shown in darker color shade. ( **C**) Schematic diagram of ligand interaction with the amino acid residues of protein during MD simulation. Interactions that occur more than 30% of the simulation time are shown.

**Figure 14.**
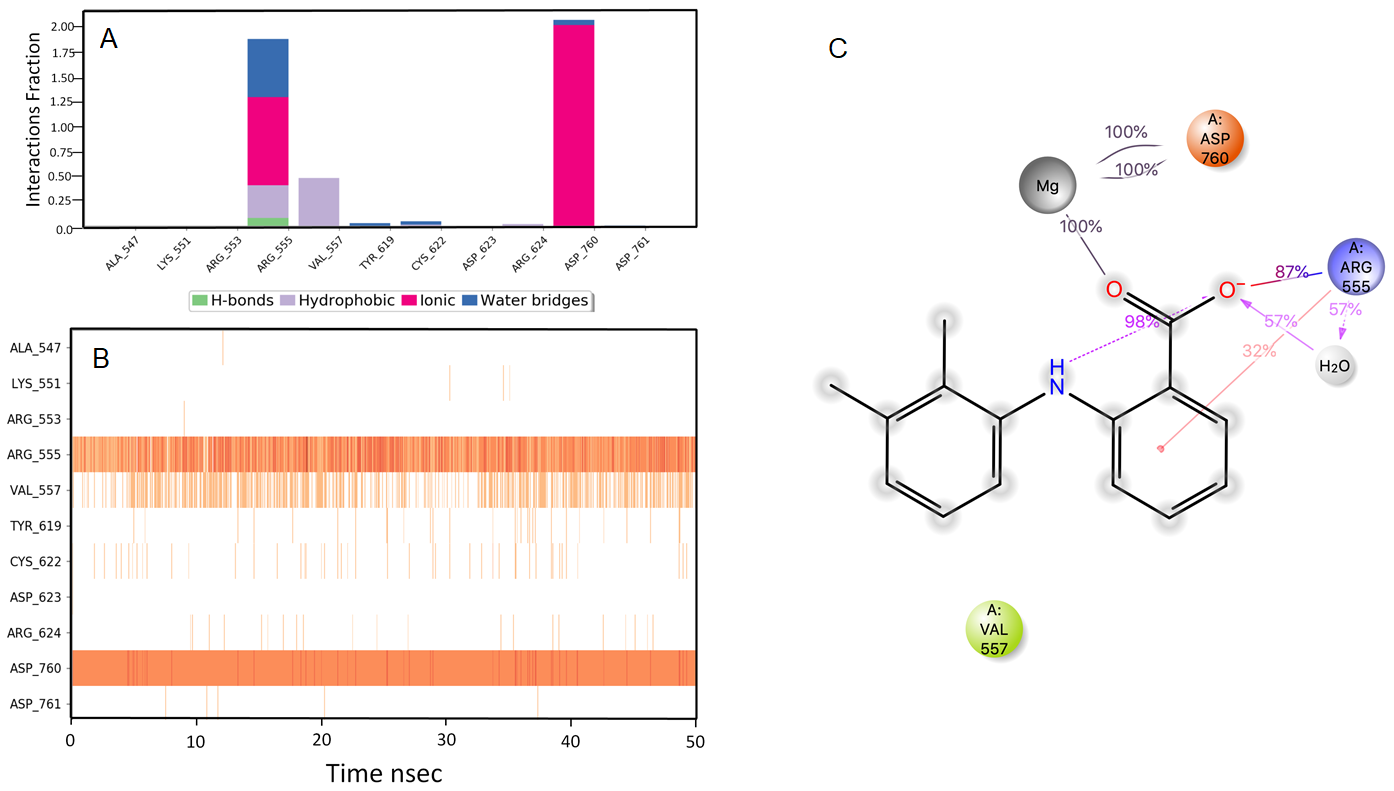
Interaction diagram of mefenamic acid with RdRp protein observed during the molecular dynamics simulation. ( **A**) The protein-ligand interaction diagram. ( **B**) The residues that interact with the ligand in each trajectory frame. The residues making more than one contact are shown in darker color shade. ( **C**) Schematic diagram of ligand interaction with the amino acid residues of protein during MD simulation. Interactions that occur more than 30% of the simulation time are shown.

**Figure 15.**
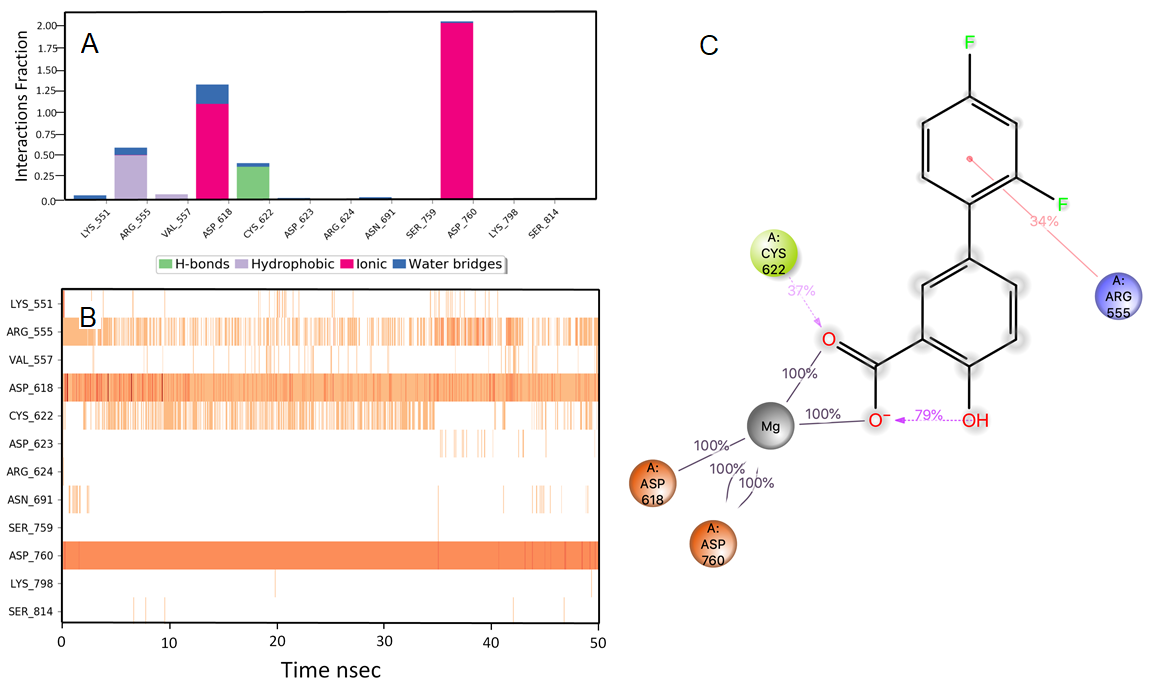
Interaction diagram of diflunisal with RdRp protein observed during the molecular dynamics simulation. ( **A**) The protein-ligand interaction diagram. ( **B**) The residues that interact with the ligand in each trajectory frame. The residues making more than one contact are shown in darker color shade. ( **C**) Schematic diagram of ligand interaction with the amino acid residues of protein during MD simulation. Interactions that occur more than 30% of the simulation time are shown.

**Figure 16.**
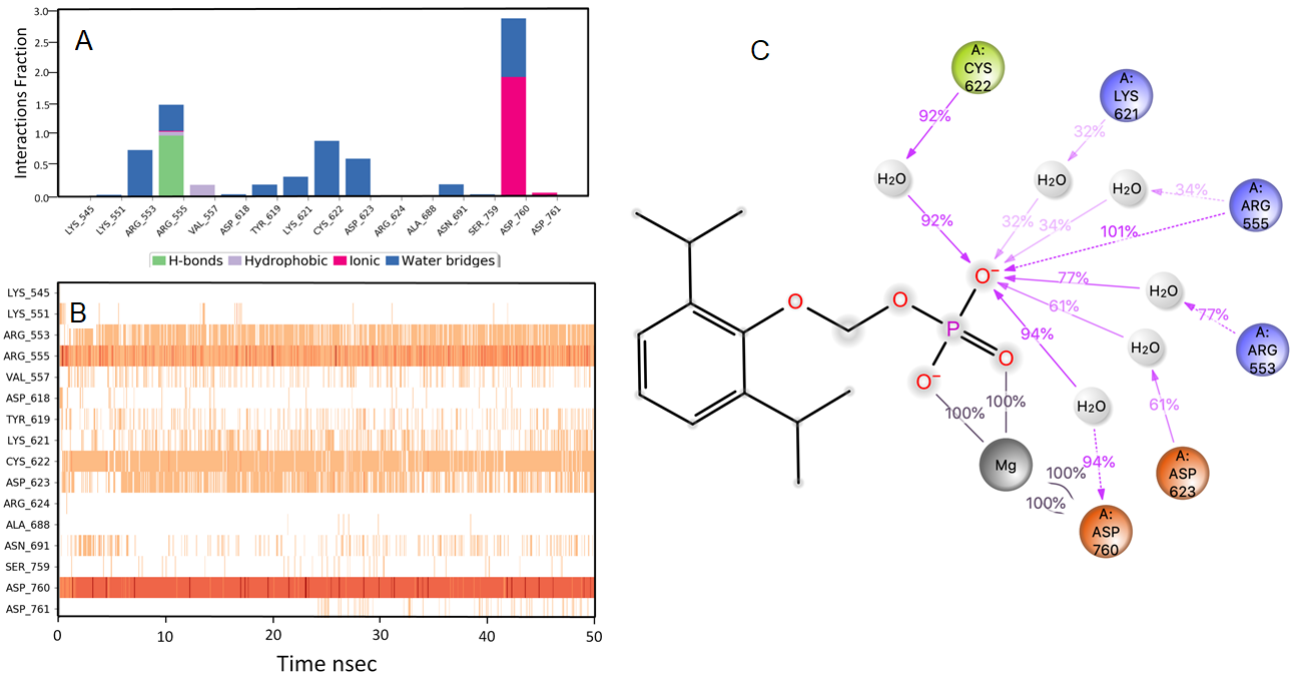
Interaction diagram of fospropofol with RdRp protein observed during the molecular dynamics simulation. ( **A**) The protein-ligand interaction diagram. ( **B**) The residues that interact with the ligand in each trajectory frame. The residues making more than one contact are shown in darker color shade. ( **C**) Schematic diagram of ligand interaction with the amino acid residues of protein during MD simulation. Interactions that occur more than 30% of the simulation time are shown.

Etodolac is an NSAID with anti-inflammatory, analgesic and antipyretic properties. It inhibits the cyclooxygenase (COX) and prevents the formation of peripheral prostaglandins which causes inflammation. It is officially prescribed for rheumatoid arthritis and osteoarthritis. Etodolac is 50-times more selective for COX-2 than COX-1, and the antipyresis is attributed to hypothalamic actions, cutaneous blood flow, and subsequent heat losses ^31^. Hence, if this drug is repurposed for COVID-19, it will have a multidirectional approach because the drugs will be useful in bringing down the elevated body temperature and anti-inflammatory properties. Montelukast, a leukotriene receptor antagonist, used to treat asthma, exercise-induced bronchoconstriction, and allergic rhinitis ^32^. The anti-inflammatory properties, as well as binding of montelukast to RdRp, might help in tackling COVID-19. Hence, montelukast can be tried for drug repurposing with detailed studies. Fluvastatin is a hypolipidemic agent belongs to the class statins ^33^. Because of their pleiotropic role, statins can be explored further to repurpose to COVID-19. Kappadione or menadiol sodium diphosphate is a highly water-soluble vitamin K analogue approved by the FDA and marketed by Lilly. It is indicated in anticoagulant-induced prothrombin deficiency, and therapy of hypoprothrombinemia due to antibacterial therapy, hypoprothrombinemia secondary to factors limiting absorption or synthesis of vitamin K. Recently, a computational study reported that kappadione would tightly bind to PAX2 transcription factor which regulates the ABC-transporter. PAX2 binding by kappadione inhibits the PAX2-DNA interaction, and this would be beneficial for combating chemoresistance in pancreatic ductal adenocarcinoma ^34^. 

Levomefolic acid, also known as 5-methyltetrahydrofolate (5-MTHF) is an active form of folate found in food, which, when administered, increases folate (vitamin B9) concentration in blood. Levomefolic acid is preferred over folic acid supplement due to lower potential for the induction of vitamin B12 deficiency symptoms ^35^. Hence, this drug is safest can be easily repurposed in COVID-19 patients. Norfloxacin is a broad-spectrum fluoroquinolone antibiotic. Norfloxacin acts by blocking DNA gyrase and used to treat UTI ^36^. Similar to quinolones, fluoroquinolones also found to modulate the cytokines, and this property is an added advantage in repurposing norfloxacin for COVID-19. Cinoxacin is currently used to treat UTIs and as an alternative antimicrobial to oxolinic acid and nalidixic acid ^37^. Tedizolid is an oxazolidinone class of antibiotics, a congener of linezolid, which is effective against multidrug-resistant Gram-positive bacteria. Tedizolid is useful in acute bacterial skin infections and was approved by the FDA in 2014. Currently available as both an oral tablet and as a powder for intravenous injection ^38^. Mefenamic acid belongs to the class of aminobenzoic acids effective for the treatment of dysmenorrhea, rheumatoid arthritis, osteoarthritis, mild to moderate pain, inflammation, and fever ^39^. Using this drug will have advantages in COVID-19, as it also tackles the inflammation and has the antipyretic effect. Diflunisal, a salicylate derivative has anti-inflammatory, analgesic, and antipyretic function as NSAID ^40^. It modulates HMGB1 through Toll-like receptor 4 (TLR4). Diflunisal does not inhibit responses that rely on TLR4. The TLR modulation is one among the targets for COVID-19, diflunisal has advantages. Fospropopol is a hypnotic/sedative/anaesthetic short-acting drug prodrug of propofol, which is an anaesthetic drug. It has a high lipid solubility can be tried in COVID-19 based on its interactions with RdRp ^41^.

Molecular docking of FDA-approved drugs for RdRp led us to shortlist 14 molecules. There were many proposals of RdRp inhibitors for repurposing, especially by *in silico* computational simulations. One previous report used the ZINC drug database to screen SARS-CoV-2 RdRp inhibitors ^13^. They studied 78 commonly used antiviral drugs and drugs which are currently in clinical trials for COVID-19 for molecular simulations and reported the possible *in silico* repurposing of drugs. They have listed the top 20 drugs from the ZINC-drug database that included the currently used antiviral drug valganciclovir, anti-bacterial drugs such as chlorhexidine, ceftibuten, cefuroxime, novobiocin, the anti-asthmatic drugs fenoterol and cromolyn, the antitumour drugs such as fludarabine, idarubicin, the antimalarial drug atovaquone, the muscle relaxant pancuronium,the anti-allergic cortisone, the contraceptive tibolone, the hepatoprotective silybin, the anti-diarrheal drug diphenoxylate, the anti-amenorrheal bromocriptine, and chenodeoxycholic acid, which is used to dissolve gallstones. In the same study, the investigators shortlisted the top-20 natural compounds as RdRp inhibitors. Another group of researchers studied the feasibility of known RNA-polymerase inhibitors to repurpose as anti-SARS-CoV-2 drugs ^42^. The drugs they studied are broad-spectrum antiviral agents such as remdesivir, 5-fluorouracil, ribavirin and favipiravir. However, they noted that the SARS-CoV-2 might evolve to acquire drug resistance mutations against these nucleoside inhibitors. The virtually combined deep learning and molecular docking simulations approach identified potentially useful 49 most promising FDA-approved drugs for repurposing in COVID-19 ^43^. The selected drugs in this study were anidulafungin, velpatasvir, glecaprevir, rifabutin, procaine penicillin G, tadalafil, riboflavin 5'-monophosphate, flavin adenine dinucleotide, terlipressin, desmopressin, elbasvir, oxatomide, enasidenib, edoxaban and selinexor. Another group of computational chemists studied the FDA-approved drugs by virtual screening for the three proteases of SARS-CoV-2, M ^pro^, PL ^pro^ and RdRp ^44^. They have shortlisted promising antiviral drugs such as simeprevir, ledipasvir, idarubicin, saquinavir, ledipasivir, partitaprevir, glecaprevir, and velpatasvir. They also shortlisted several novel drugs such as antiviral raltegravir; antidiabetic amaryl, antibiotics retapamulin, rifimixin, and rifabutin; antiemetic fosaprepitant and netupitant. Another group of researchers used a multi-targetted approach and shortlisted molecules such as δ-viniferin, myricitrin, taiwanhomoflavone A, lactucopicrin 15-oxalate, nympholide A, afzelin, biorobin, hesperidin and phyllaemblicin B. These molecules have the affinity for SARS-CoV-2 M ^pro^, RdRp and hACE2 ^45^. Furthermore, a group of researcher have proposed well-tolerated, cost-effective drugs to combat COVID-19 which has the affinity for both M ^pro^ and RdRp. The compounds they have shortlisted are ergotamine, dihydroergotamine, conivaptan, paliperidone, and tipranavir ^46^.

In the pathogenesis and pathophysiology of COVID-19, both innate and adaptive immunity are activated, and many inflammatory mediators are released ^47^. Mitigating inflammation will reduce the lethality because death due to COVID-19 is partly due to cytokine storm and end-organ failure ^48– 50^. The immuno-inflammatory modulators have their role in the treatment of COVID-19, and the drug shortlisted in this study have those potentials have potentials to mitigate inflammation. The shortlisted 14 novel molecules for SARS-CoV-2 RdRp inhibitors from different pharmacological classes. These drugs include antimicrobials such as rosoxacin, norfloxacin, cinoxacin, tedizolid phosphate, vitamin analogues such as kappadione, levomefolic acid, anti-inflammatory agents such as etodolac, mefenamic acid, diflunisal, montelukast, statins such as pitavastatin, fluvastatin, a sedative fospropofol, and a thromboxane inhibitor ridogrel. These drugs are commonly used, well-tolerated and economical. Among the 14-drugs, pitavastatin, is a lipid lowering agent and has pleotropic effects. Ridogrel rosoxacin, a quinolone antibiotic indicated for the treatment of urinary tract infections and certain sexually transmitted diseases, Testing *in vitro* for RdRp inhibitory activity, and antiviral activity could confirm these molecules for the repurposing or repositioning for COVID-19 treatment.

## Conclusion

In the absence of approved therapies for treatment or prevention, drug repurposing has provided valuable insight into the treatment of COVID-19. The antiviral drugs such as lopinavir, ritonavir, favipiravir and remdesivir, immunosuppressants such as sirolimus, anthelmintics like ivermectin, corticosteroids such as methylprednisolone, dexamethasone are currently under clinical trials for COVID-19. In this molecular docking study using structure-based virtual screening, we identified favourable drugs, namely rosoxacin, levomefolic acid, etodolac, kappadione, pitavastatin, montelukast, fluvastatin, norfloxacin, cinoxacin, tedizolid phosphate, mefenamic acid, diflunisal, fospropofol, and ridogrel. The MD simulation studies revealed pitavastatin, ridogrel and rosoxacin to be superior compared to other shortlisted drugs. Further, the therapeutic properties of these shortlisted molecules make it suitable for testing RdRp-inhibitory activity and antiviral activity *in vitro* and *in vivo* could confirm these molecules for repurposing in COVID-19.

## Data availability

All data underlying the results are available as part of the article and no additional source data are required.
