# Supplementary material for: Targeting SARS-CoV-2 RNA-dependent RNA polymerase: An in silico drug repurposing for COVID-19
Source: F1000Res. 2020 Sep 23;9:1166. [Version 1] doi: 10.12688/f1000research.26359.1 (PMC7610171; doi:10.12688/f1000research.26359.1)
Supplement: Peer review history

# Peer review: Reviewer response for version 1

**DOI:** 10.5256/f1000research.29098.r71828

**Published:** 2020-11-03

**Authors:** Hemachandra Reddy

**Affiliations:** 1 Garrison Institute on Aging, Texas Tech University Health Sciences Center, Lubbock, TX, USA

It is a nicely done work, used methods are acceptable - used computational biology is acceptable - Authors shortlisted 14 drugs from the Standard Precision docking and interaction-wise study of drug-binding with the active site on the enzyme. These drugs are antibiotics, NSAIDs, hypolipidemic, coagulant, thrombolytic, and anti-allergics. In molecular dynamics simulations, pitavastatin, ridogrel and rosoxacin displayed superior binding with the active site through ARG555 and divalent magnesium. It is timely and current version is ready to be indexed.

Is the work clearly and accurately presented and does it cite the current literature?

Yes

If applicable, is the statistical analysis and its interpretation appropriate?

Not applicable

Are all the source data underlying the results available to ensure full reproducibility?

Yes

Is the study design appropriate and is the work technically sound?

Yes

Are the conclusions drawn adequately supported by the results?

Yes

Are sufficient details of methods and analysis provided to allow replication by others?

Yes

Reviewer Expertise:

Inflammation, computational biology, neurodegeneration, aging and Alzheimer's disease.

I confirm that I have read this submission and believe that I have an appropriate level of expertise to confirm that it is of an acceptable scientific standard.

# Peer review: Reviewer response for version 1

**DOI:** 10.5256/f1000research.29098.r71827

**Published:** 2020-10-23

**Authors:** Harish Holla

**Affiliations:** 1 Department of Chemistry, Central University of Karnataka, Kalaburagi, India

An *in silico* drug repurposing work is being reported by authors targeting against COVID-19. Authors have attempted to find the possibility of finding drug in the repository of available drug bank which can be repurposed.

 Many of the articles are appearing where computational tools have been utilized targeting the major protease (M ^pro^), and RNA-dependent RNA polymerase (RdRp) which are two validated target proteins till now.

 Authors have docked the FDA approved library of drugs against the active site of RdRp using Schrodinger's computer-aided drug discovery tools for *in silico* drug-repurposing. They have shortlisted 14 drugs from the Standard Precision docking and interaction-wise study of drug-binding with the active site on the enzyme. These drugs are antibiotics, NSAIDs, hypolipidemic, coagulant, thrombolytic, and anti-allergics. Also, they have carried out MD simulation studies on the best among those. The study will be useful for further explorations for *in vitro* and *in vivo *studies. The authors have focused only on computational studies and *in vitro* studies on selected few would have been handy. 

 Overall, the work is well drafted with minor errors and is suitable for the researchers who are looking for molecules to be studied *in vitro* on COVID-19.

Is the work clearly and accurately presented and does it cite the current literature?

Yes

If applicable, is the statistical analysis and its interpretation appropriate?

I cannot comment. A qualified statistician is required.

Are all the source data underlying the results available to ensure full reproducibility?

Yes

Is the study design appropriate and is the work technically sound?

Yes

Are the conclusions drawn adequately supported by the results?

Yes

Are sufficient details of methods and analysis provided to allow replication by others?

Yes

Reviewer Expertise:

Synthetic Organic Chemistry, Medicinal chemistry, Natural Product Chemistry.

I confirm that I have read this submission and believe that I have an appropriate level of expertise to confirm that it is of an acceptable scientific standard.
